# Highly sensitive tryptophan fluorescence probe for detecting rhythmic conformational changes of KaiC in the cyanobacterial circadian clock system

**DOI:** 10.1042/BCJ20210544

**Published:** 2022-07-19

**Authors:** Atsushi Mukaiyama, Yoshihiko Furuike, Eiki Yamashita, Shuji Akiyama

**Affiliations:** 1 Research Center of Integrative Molecular Systems (CIMoS), Institute for Molecular Science, National Institutes of Natural Sciences, 38 Nishigo-Naka, Myodaiji, Okazaki 444-8585, Japan; 2 Department of Functional Molecular Science, SOKENDAI (The Graduate University for Advanced Studies), 38 Nishigo-Naka, Myodaiji, Okazaki 444-8585, Japan; 3 Institute for Protein Research, Osaka University, 3-2 Yamada-oka, Suita 565-0871, Japan

**Keywords:** circadian clock, cyanobacteria, KaiC, solution structural change, Trp fluorescence

## Abstract

KaiC, a core protein of the cyanobacterial circadian clock, consists of an N-terminal CI domain and a C-terminal CII domain, and assembles into a double-ring hexamer upon binding with ATP. KaiC rhythmically phosphorylates and dephosphorylates its own two adjacent residues Ser431 and Thr432 at the CII domain with a period of ∼24 h through assembly and disassembly with the other clock proteins, KaiA and/or KaiB. In this study, to understand how KaiC alters its conformation as the source of circadian rhythm, we investigated structural changes of an inner-radius side of the CII ring using time-resolved Trp fluorescence spectroscopy. A KaiC mutant harboring a Trp fluorescence probe at a position of 419 exhibited a robust circadian rhythm with little temperature sensitivity in the presence of KaiA and KaiB. Our fluorescence observations show a remarkable environmental change at the inner-radius side of the CII ring during circadian oscillation. Crystallographic analysis revealed that a side chain of Trp at the position of 419 was oriented toward a region undergoing a helix–coil transition, which is considered to be a key event to allosterically regulate the CI ring that plays a crucial role in determining the cycle period. The present study provides a dynamical insight into how KaiC generates circadian oscillation.

## Introduction

The circadian clock is an endogenous time-management system that rhythmically regulates biological activities to adapt to external periodic environmental changes. Since the discovery of a gene involved in the circadian clock in Drosophila [1], many clock genes have been identified in various species including mammals [2], plants [3], fungi [4] and prokaryotes [5]. These findings led to the proposal of the transcription–translation feedback loop (TTFL) model as a common molecular mechanism behind the circadian rhythm *in vivo*, in which clock proteins, translation products of the clock genes, suppress their own gene expression [6]. Recent studies, however, have reported the existence of non-transcriptional circadian rhythms generated by post-translational modifications of the clock proteins [7,8], which is considered to play a key role in the circadian timekeeping mechanism. Thus, it is particularly important to uncover the dynamic structural changes of the clock proteins as a source of the oscillation.

The circadian clock of a cyanobacterium *Synechococcus elongatus* PCC 7942 has been extensively studied to elucidate the structures and functions of clock proteins [9,10]. The core oscillator consists of the three kinds of proteins, KaiA, KaiB and KaiC and the clock can be reconstructed *in vitro* by mixing the three Kai-proteins with ATP [11]. KaiC, a core protein of the clock, consists of two tandemly duplicated domains, an N-terminal (CI) domain and a C-terminal (CII) domain, and forms a hexamer upon binding with ATP [12]. KaiC periodically recruits and releases KaiA and/or KaiB [13,14] through its ATP-hydrolysis, kinase and phosphatase reactions [15–17]. The post-translational phosphorylation and dephosphorylation occur at S431 and T432 in the CII domain. When co-incubated with KaiA and KaiB, KaiC exhibits circadian phosphorylation cycle as follows: ST → SpT → pSpT → pST → ST, where S, T, pS and pT represent S431, T432, phosphorylated S431 and phosphorylated T432, respectively [18,19]. The period length of the cycle is almost kept constant over physiological ranges of temperature. This is a common feature of the circadian clock (temperature-compensation) [20]. Although the KaiC phosphorylation cycle has been extensively examined [21,22], it is not well understood how KaiC changes its structure in real time in solution.

**Figure 1.**
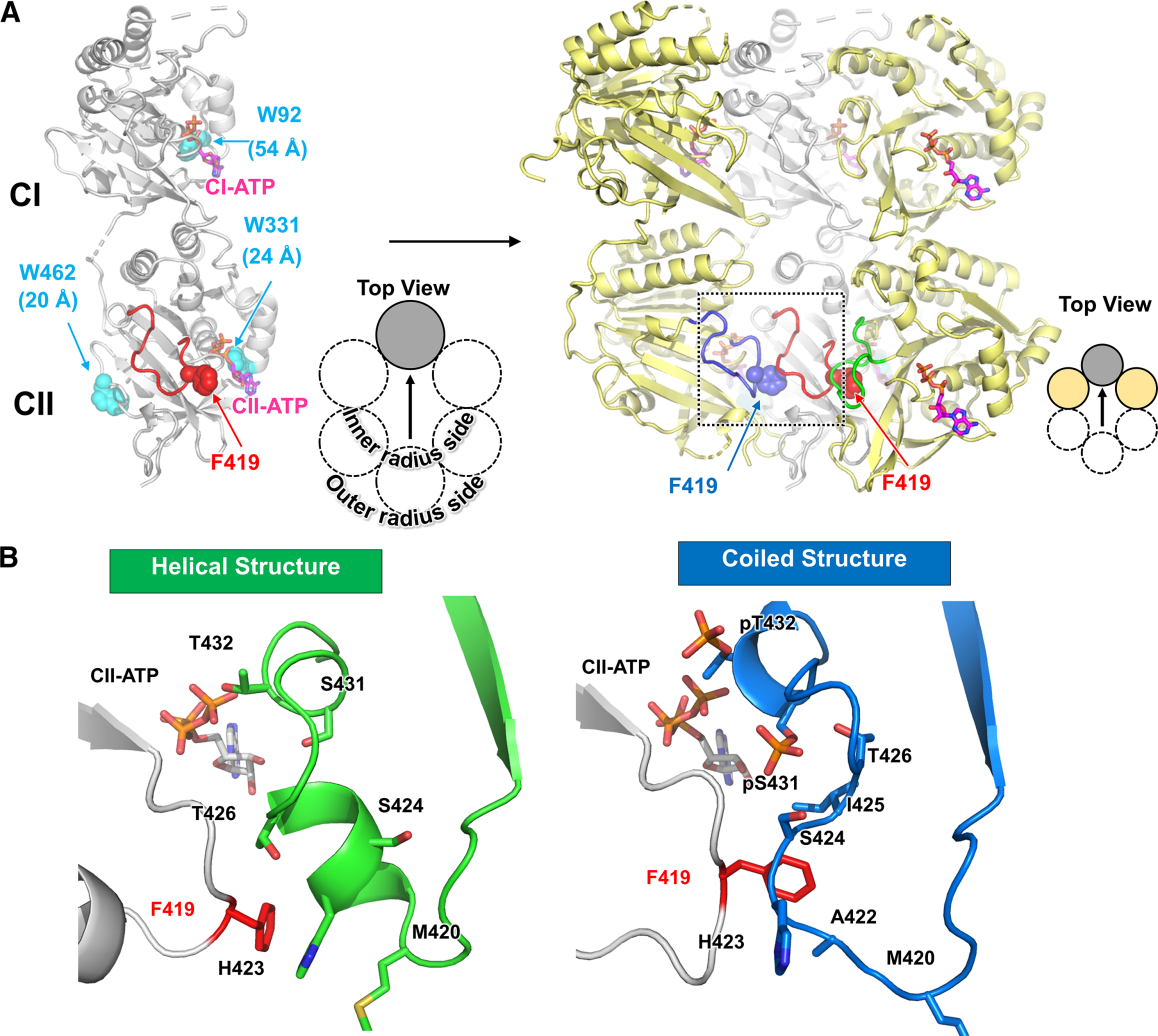
Design of KaiC^F419W^ mutant. (**A**) Mapping of intrinsic Trp residues (W92, W331 and W462) and F419 on one or three protomers in KaiC hexamer, which is viewed from the inner-radius side of the hexameric ring. The values below the intrinsic Trp residues (W92, W331 and W462) indicate the distances from F419. The dashed box is the region corresponding to (**B**). (**B**) Comparison of two conformations found in the crystal structures [29] at the inner-radius side of the CII ring. The side chains of F419 and the amino acid residues from the neighboring protomer are depicted by stick models.

Trp fluorescence spectroscopy is widely used to investigate the structural changes of proteins in solution because the emission from Trp fluorophore is highly sensitive to its surrounding environment [23–26]. We have previously shown that this method is quite useful to study the Kai-protein clock system. Wild-type KaiC (KaiC^WT^) intrinsically has three Trp residues, one (W92) at the CI domain and two (W331 and W462) at the CII domain (Figure 1A). In the presence of KaiA and KaiB, an fluorescence emission from W331 and/or W462 in KaiC is rhythmically altered during the phosphorylation cycle [27]. On the other hand, the emission from W92 is almost insensitive to the structural changes of the CI ring, but we have revealed the CI-ring rearrangement coupled with ATPase and phosphorylation state by introducing exogenous Trp residues on the CI domain [28]. These observations indicate that fluorescence spectroscopy combined with Trp mutagenesis is a powerful tool to uncover the dynamic structural changes of KaiC in solution.

In this study, we focused on an inner-radius side of the CII ring in KaiC as the research target based on our recent observation of KaiC crystal structures, in which an upstream region of the phosphorylation sites (T416-H429) at the inner-radius side undergoes a structural change [29]. Using a KaiC mutant harboring an additional Trp at a position of F419 (KaiC^F419W^), we probed rhythmic structural changes at an inner-radius side of the CII ring during circadian oscillation. Through the present study, we propose the usefulness of Trp-based fluorescence spectroscopy for studying the circadian structural changes of the clock proteins in real time.

## Results

### Construction and characterization of KaiC^F419W^

**Figure 2.**
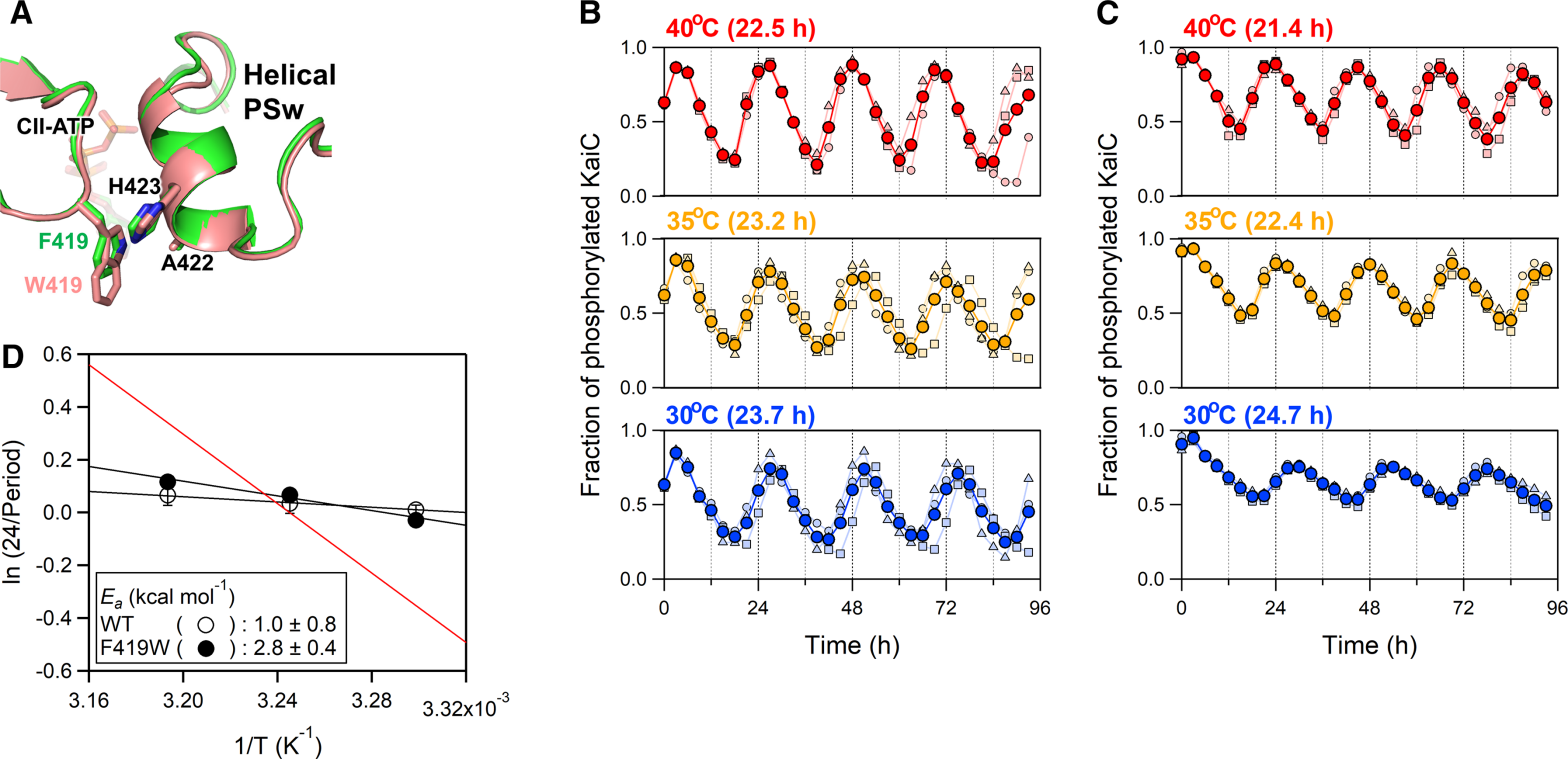
Structural and functional intactness of KaiC^F419W^. (**A**) Zoomed-in view of an inner-radius side of the CII ring in KaiC^F419W^ (pink subunits, accession code: 7WDC) superimposed onto KaiC^WT^ (green subunits, accession code: 7DYJ). The phosphorylation cycle of KaiC^WT^ (**B**) and KaiC^F419W^ (**C**) at different temperatures. Blue, orange and red markers represent the fractions of the phosphorylated KaiC at 30, 35 and 40°C, respectively. Each filled circle represents the mean from three independent measurements (pale-colored squares, triangles and diamonds). (**D**) Arrhenius plot analysis of phosphorylation cycle frequencies (24/period) for KaiC^WT^ and KaiC^F419W^. The activation energy (*E_a_*), which was determined by linear regression (solid lines). Red line represents the result of a hypothetical rhythm with a period of 24 h and a *Q*_10_ value of 2 at 30°C (*E_a_ *= 13.1 kcal mol^−1^) as seen in a canonical chemical reaction.

According to the crystal structures of KaiC [29] (Figure 1B), the side chain of F419 is oriented toward A422 and H423 on the neighboring protomer. We thus inserted a Trp residue at position 419 (KaiC^F419W^) to monitor the conformational change around the inner-radius side in solution by fluorescence spectroscopy. The F419W substitution had little impact on the overall structure of KaiC as evidenced by backbone RMSD of 0.42 Å between KaiC^F419W^ and KaiC^WT^ (Figure 2A). Moreover, our biochemical assay demonstrated that KaiC^F419W^ is virtually indistinguishable from KaiC^WT^ in terms of clock function (Figure 2B,C). In the presence of KaiA and KaiB, KaiC^F419W^ rhythmically changed its phosphorylation level with a period of 24.7 ± 0.3 h (24.7 ± 0.4 h for KaiC^WT^) at 30°C. The period length of the phosphorylation cycle was less dependent on temperatures and the *Q*_10_ value at 30°C was estimated to be 1.15 ± 0.02 (1.07 ± 0.03 for KaiC^WT^) from the Arrhenius plot as shown in Figure 2D.

### W419 fluorescence as a probe for detecting circadian rhythms

**Figure 3.**
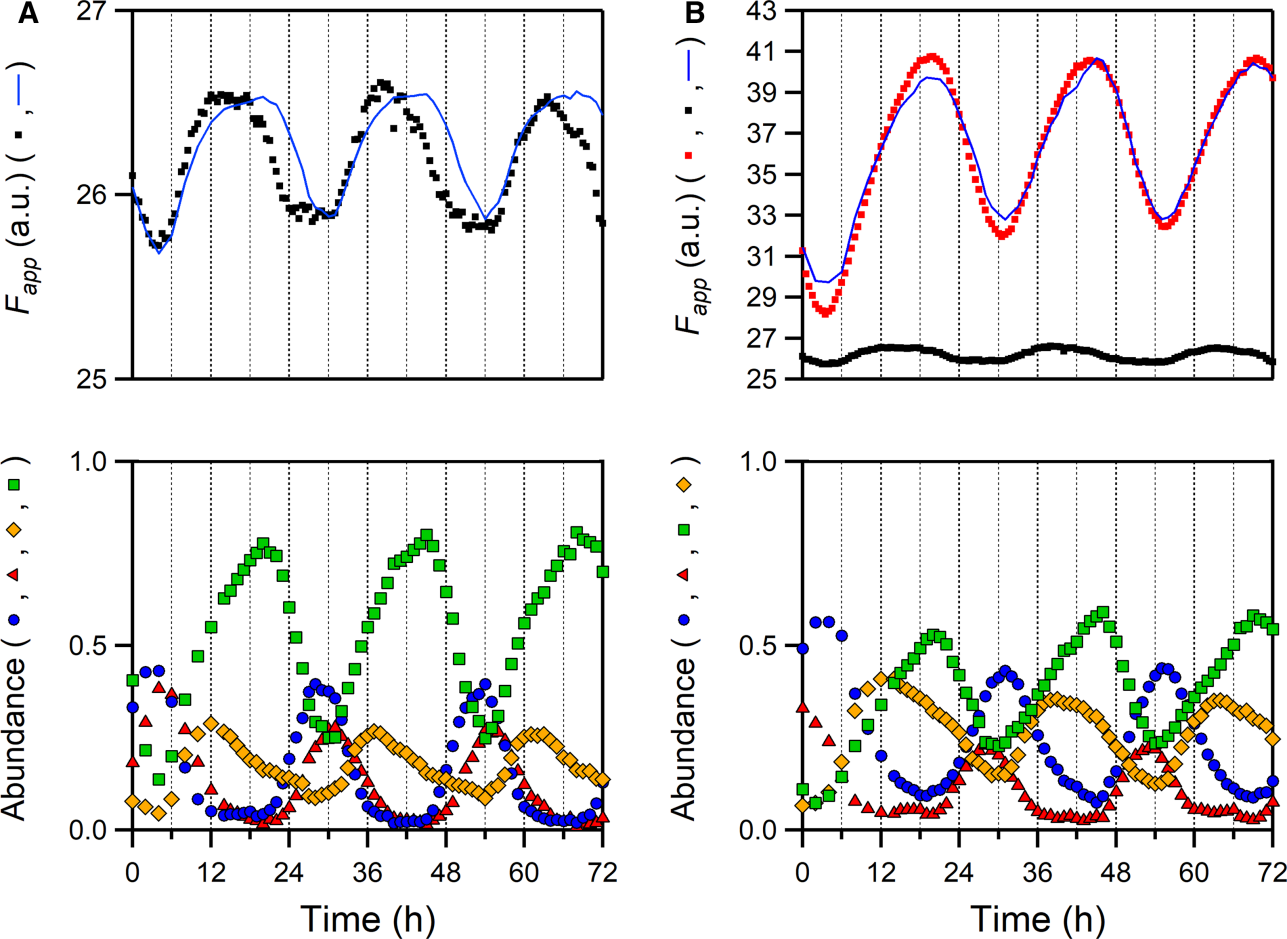
Time-courses of fluorescence intensity (*F*) of KaiC^WT^ (A) and KaiC^F419W^ (B) during circadian oscillation. (*Upper*) The apparent *F* (*F*_app_) changes of KaiC^WT^ (black dots) or KaiC^F419W^ (red dots) in the presence of KaiA and KaiB. The blue solid lines in (**A**,**B**) represent the results simulated based on equation (1) by using *F* values shown in Figure 4C and *A_i_*(*t*) shown in the lower panel of respective figures. Each plot includes an offset of 19.8 in (**A**) and 16.0 in (**B**), respectively, which likely originates from the contribution of KaiA fluorescence. (*Lower*) Temporal patterns of relative abundance of the SpT (red triangle), pSpT (blue circle), pST (orange diamond) and ST (green square) states of KaiC^F419W^.

First, we performed time-resolved Trp fluorescence measurements of solutions containing KaiA, KaiB and KaiC (KaiC^WT^ or KaiC^F419W^). Trp fluorescence spectra were measured using an excitation wavelength of 295 nm and the fluorescence intensity integrated from 320 to 370 nm (*F*_app_) was recorded every 30 min. As shown in the upper panels of Figure 3A,B, *F*_app_ values of solutions containing KaiC^WT^ or KaiC^F419W^ changed rhythmically; the oscillations lasted at least for 4 days without apparent damping. The amplitude of *F*_app_ oscillation, the difference between the maximum and minimum florescence signal, was enhanced from 0.6 to 8.1 by F419W substitution. A significant increase in the amplitude indicates that the oscillation in *F*_app_ of KaiC^F419W^ largely reflects a rhythmic change in the environment surrounding the W419 site in solution. When the time-courses of *F*_app_ values were compared with those of relative abundance in the four phosphorylation states (lower panels in Figure 3A,B), the phase with the maximum *F*_app_ value of KaiC^WT^ roughly coincided with the phase at which KaiC-pST was maximally populated as reported previously [27]. On the other hand, the phases of maximum and minimum *F*_app_ values of KaiC^F419W^ matched the phases where KaiC-ST and KaiC-pSpT states were maximally accumulated, respectively. This result suggests that the sensitivity of W419 fluorescence changes rhythmically in a phase-dependent manner but different from that of the intrinsic Trp residues.

### Phosphoryl modification at S431 alters the conformation at the inner-radius side of the CII ring

**Figure 4.**
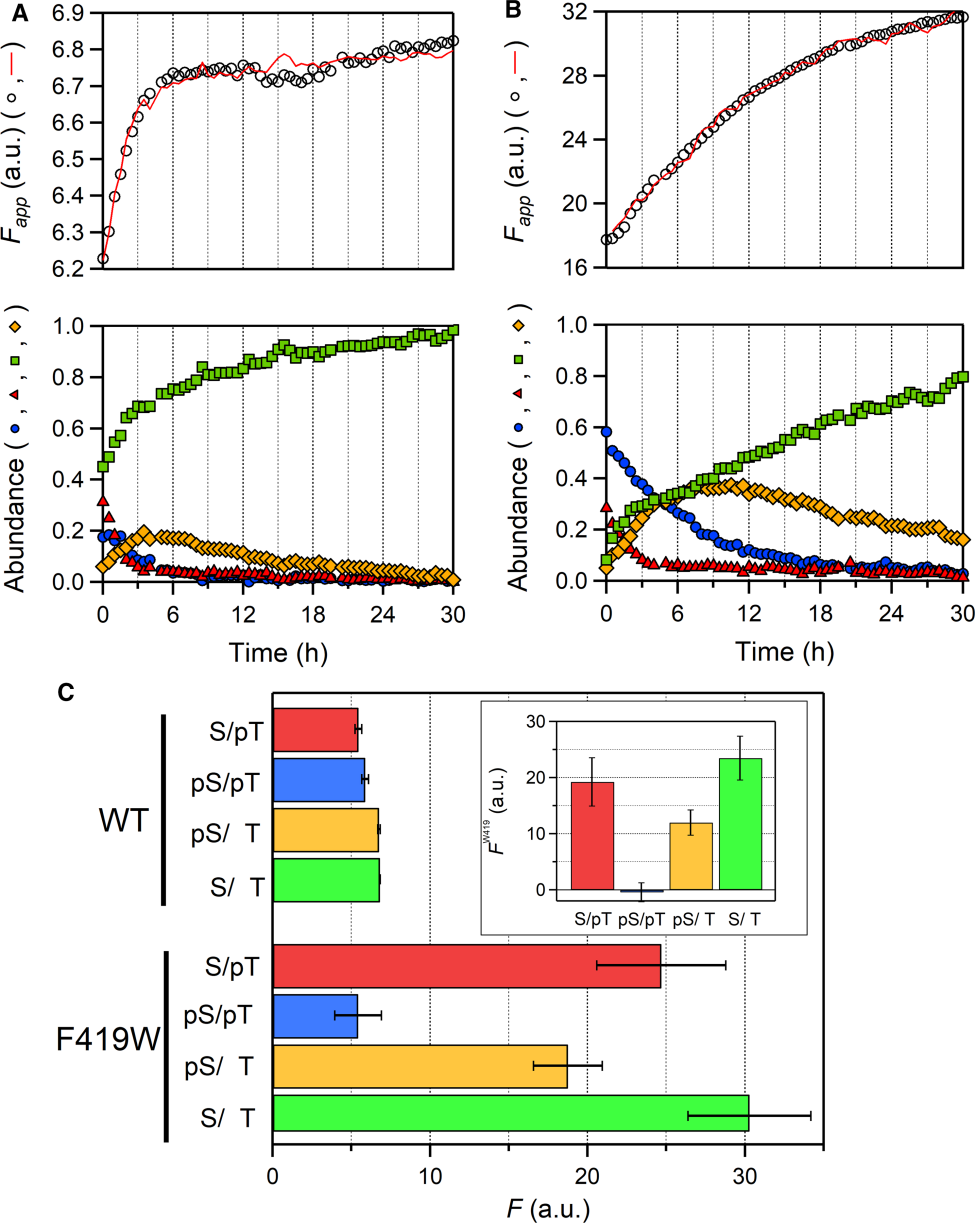
Change of W419 fluorescence during the auto-phosphorylation process. (*Upper*) Time-courses of *F*_app_ (open circles) of KaiC^WT^ (**A**) and KaiC^F419W^ (**B**) during auto-dephosphorylation. Red solid lines represent the results of fitting using equation (1). (*Lower*) Temporal patterns of relative abundance of the SpT, pSpT, pST and ST states. Color indication about the phosphorylation state is the same as shown in Figure 3. (**C**) *F* for KaiC^WT^ and KaiC^F419W^ as a function of the phosphorylation state Each value represents the mean ± S.D. from three independent measurements. Inset shows W419 fluorescence as a function of the phosphorylation state. The contribution of W419 fluorescence (*F*^W419^) was estimated as the difference between KaiC^WT^ and KaiC^F419W^ at each phosphorylation state.

Since KaiA also intrinsically possesses a Trp residue (W10), the amplitude of *F*_app_ oscillation of a solution containing KaiA, KaiB and KaiC^F419W^ (Figure 3) might include the contribution from KaiA. To determine the extent to which the fluorescence from W419 was altered upon the structural change of KaiC itself, we next carried out time-resolved fluorescence measurements during the auto-dephosphorylation process of KaiC alone. Auto-dephosphorylation of KaiC was initiated by transferring a solution containing KaiC^WT^ or KaiC^F419W^ from an ice bath to 30°C. As reported previously [27,28], the *F*_app_ value of KaiC^WT^ was gradually increased concomitant with a change in the relative abundance of the four phosphorylation states (Figure 4A). *F*_app_ of KaiC^F419W^ also showed a gradual increase but with the 25-fold larger amplitude than that of KaiC^WT^ (Figure 4B).

Then, we estimated the *F* value of each phosphorylation state (*F*_i_) by assuming that *F*_app_(*t*) is represented as a linear summation of the contributions from four phosphorylation states as reported previously [28] (see details in Materials and methods). Interestingly, the *F* value of KaiC^F419W^ at the SpT, pST and ST states was much larger than that of KaiC^WT^ at the respective states. On the other hand, the *F* value of KaiC^F419W^ at the pSpT state was comparable to that of KaiC^WT^ (Figure 4C). As reported in our previous paper [27], simulated rhythmic changes in the *F* value (solid blue line in Figure 3A) using the *F* values of KaiC^WT^ (Figure 4C) and relative abundance during circadian oscillation (Lower panel in Figure 3A) were coincident with the observed temporal pattern during the phosphorylation phase and deviated during the dephosphorylation phase. For KaiC^F419W^, better coincidence between the observed and simulated *F* values was observed throughout the whole cycle. These results demonstrate that the fluorescence of KaiA during the oscillation can be interpreted as the constant signal and its contribution to the cyclic fluorescence change is negligibly small.

Using these *F* values, we extracted the fluorescence contributions from W419 (*F*^W419^) at each phosphorylation state by subtracting the *F* value of KaiC^WT^ from that of KaiC^F419W^ (Figure 4C, inset). As a result, the fluorescence emission of W419 was significantly quenched in the transition from KaiC-SpT to KaiC-pSpT and then dequenched in a stepwise manner during the transition from KaiC-pSpT to KaiC-ST via KaiC-pST. On the other hand, there was a minor difference in *F*^W419^ between KaiC-ST and KaiC-SpT. These results suggest that phosphorylation at S431 leads to the conformation change around an inner-radius side of the CII ring.

### Phospho-dependent alteration in the polarity of W419

**Figure 5.**
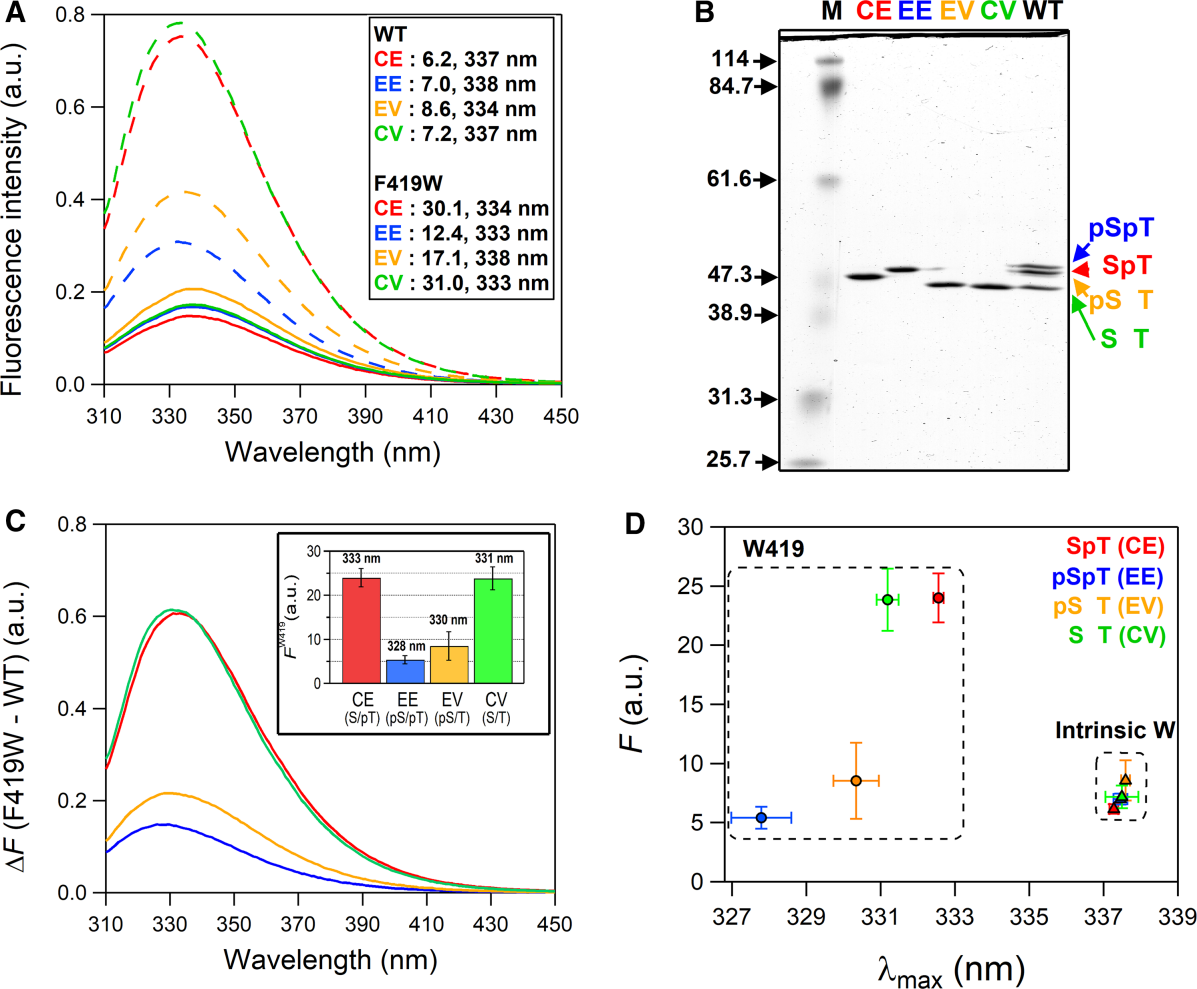
Trp fluorescence characterization of phospho-mimicking mutants. (**A**) Trp fluorescence spectra of CE (red), EE (blue), EV (orange) and CV (green) mutants with (dashed lines) and without (solid line) the F419W substitution. Values shown in parentheses represent the fluorescence intensity and maximum fluorescence wavelength of each mutant, respectively. (**B**) SDS-images of KaiC^WT^ and its phospho-mimicking mutants. Lane M represents the molecular mass marker. (**C**) Difference spectra of CE, EE, EV and CV with or without the F419W substitution. Color indication for each mutant is the same as shown in (**A**). Inset shows fluorescence intensities from W419 (*F*^W419^) of CE, EE, EV and CV. (**D**) Two-dimensional plot between the fluorescence emission maximum (*λ*_max_) and fluorescence intensity (*F*) for CE, EE, EV and CV. Circles and triangles represent data for fluorescence of intrinsic Trps (W92, W331 and W462) and W419, respectively. Each value represents the mean ± S.D. from three independent measurements.

To further characterize the structural change, we performed static Trp fluorescence measurements using a series of mutants mimicking each phosphorylation state (Figure 5A). To minimize the structural perturbation upon mutations, we selected non-phosphorylation amino acids in terms of side-chain volumes and topologies, and replaced S431, T432, and phosphorylated residues with cysteine, valine and glutamate as follows: an SpT-mimicking S431C/T432E mutant (KaiC-CE), a pSpT-mimicking S431E/T432E (KaiC-EE), a pST-mimicking S431E/T432V (KaiC-EV) and an ST-mimicking S431C/T432V mutant (KaiC-CV). We confirmed by SDS–PAGE analysis that each designed phospho-mimicking mutant showed a similar migration pattern to the corresponding phospho-form in KaiC^WT^ (Figure 5B). Figure 5C shows difference spectra of phospho-mimicking mutants with and without F419W substitution. The fluorescence contributions from W419 (*F*^W419^) of each phospho-mimicking mutant were extracted by subtracting *F* of each mutant without F419W substitution from that with the substitution (Figure 5C, inset). The variation in *F*^W419^ among phospho-mimicking mutants was similar to that obtained by the deconvolution analysis (Figure 4C, inset), further supporting that the observed *F*_app_ changes during auto-dephosphorylation (Figure 4B) as well as the circadian oscillation (Figure 3B) originate from the interconversion among four phosphorylation states in solution.

In addition to the fluorescence intensity, the fluorescence emission maximum (λ_max_) was shifted depending on the phosphorylation states (Figure 5C). The λ_max_ is frequently used as a measure of hydrophobicity around Trp environment. For example, when a Trp residue is exposed to the solvent upon protein denaturation, λ_max_ is red-shifted [30,31]. Figure 5D shows a two-dimensional plot between *F* and λ_max_ for the phospho-mimicking mutants. The data points corresponding to W419 fluorescence are placed in a bottom area on the *F–*λ_max_ plot as compared with those including the fluorescence contribution from the other three intrinsic Trp residues, suggesting that W419 is situated in a more hydrophobic environment than the intrinsic Trp residues. More interestingly, significant state-dependent variations were observed not only in fluorescence intensity but also in λ_max_ of W419 fluorescence. These observations demonstrate that the inner-radius side of the CII ring undergoes a substantial structural transition associated with remarkable alteration in hydrophobicity in accordance with the phosphorylation state.

## Discussion

In this study, we examined the structural change of the inner-radius side of the CII ring by detecting the fluorescence emission from the Trp residue replaced with F419 (KaiC^F419W^). KaiC^F419W^ showed robust circadian rhythm with the *Q*_10_ value of 1.15 ± 0.02 (Figure 2C). Temperature-compensation is a hallmark of the circadian clock system and it has been reported that the *Q*_10_ values of the circadian rhythms seen in diverse organisms range from 0.8 to 1.4 [32]. Furthermore, we have found several KaiC mutants with the *Q*_10_ value above 1.4 [33]. Given these observations, it is reasonable to conclude that KaiC^F419W^ retains the temperature-compensation property. The F419W substitution resulted in substantial increases in the amplitudes of *F* change during both circadian oscillation (Figure 3) and auto-dephosphorylation (Figure 4) processes. These observations demonstrate that the inner-radius side of the CII ring undergoes a structural change in the solution.

W419 fluorescence of the ST state was comparable to that of the SpT state, but was almost completely quenched in the transition from the SpT to pSpT states and then dequenched from the pSpT to ST via pST states (Figure 4C, inset). The observed quenching/dequenching is associated with the shift in the fluorescence emission maximum (λ_max_) as observed by static fluorescence spectra of phospho-mimicking mutants (Figure 5C). Notably, the range of the state-dependent variation in λ_max_ of W419 fluorescence (∼5 nm) was wider than that of the intrinsic Trp residues (∼1 nm), including large contributing W462 [27]. This suggests that the environment around W419 is altered more significantly than that of W462.

In our previous report [28], a drastic fluorescence change comparable to that of W419 was observed when a Trp probe (W146) was introduced into the site where *cis*-*trans* isomerization of D^145^S^146^ peptide occurs during ATP-hydrolysis in the CI domain. In addition, the fluorescence emission from the three intrinsic Trp residues showed an obvious but slight change in response to the phosphorylation state, whereas it increased by about 5-fold upon dissociation into KaiC monomers concomitant with a red-shift in λ_max_ by ∼4 nm. Although the global conformational changes of the CII ring upon S431 phosphorylation have been reported previously [27,34], the present results indicate a local but remarkable structural change that occurs even at the inner-radius side in solution.

According to the recently solved crystal structures of KaiC at four distinct phosphorylation states [29], an upstream region of the two phosphorylation sites (T416-S429) at the inner-radius side undergoes structural transitions (Figure 1B), which are key motions for the oscillatory nature of KaiC. The region adopted a coil structure in KaiC-pSpT and KaiC-pST, while a helical structure was observed in both KaiC-ST and a KaiC-SpT mimicking mutant KaiC-T432E. Crystal structure of KaiC^F419W^-ST (Figure 2A) revealed that the indole ring of W419 was oriented toward the helical structure on the neighboring protomer. This suggests that the phospho-dependent variation of W419 fluorescence reflects the environmental change associated with the helix–coil transition. Although an interpretation of fluorescence from tryptophan in proteins is a challenging issue, one of the potential quenchers of W419 fluorescence on the basis of crystal structures of KaiC is a sulfur atom of M420 [35]. The side chain of W419 (or F419) is far from M420 in the ST state where the helical structure is formed, whereas they are in close proximity each other in the pSpT and pST states, in which the upstream region adopts the coil structure. The difference in W419 fluorescence emission between the pSpT and pST states suggests that the inner-radius side of the CII ring adopts alters a conformation while the coiled structure is kept during the auto-dephosphorylation process from the pSpT to pST.

The main advantage of the Trp fluorescence method is that, in principle, the probe can be introduced anywhere in the amino acid sequence in proteins. In this study, we took full advantage of the method and succeeded in detecting local structural changes at the inner-radius side of KaiC hexamer in real time, which are often inaccessible by probes targeting global conformational changes. On the other hand, a variety of crystal structures of KaiC reported so far have provided information on the structural transition at atomic resolution [12,29]. An integrated approach of Trp fluorescence spectroscopy and crystallography will deepen our understanding of KaiC, the core protein of the cyanobacterial circadian clock.

## Materials and methods

### Protein expression and purification of Kai-proteins

All plasmid vectors used in this study were generated for glutathione S-transferase (GST)-tagged (pGEX-6P-1) form [36]. The genes for *kaiC* mutants were all synthesized and incorporated into pGEX-6P-1 vector containing *kaiC^WT^* using *Sac*I/*Eco*RI sites by Eurofin Genomics. The synthesized bases are shown in Supplementary Figure S1. Recombinant Kai-proteins were expressed in *E. coli* and purified as reported previously [36].

### Biochemical assays of Kai-proteins

All measurements were conducted in a buffer containing 50 mM Tris–HCl (pH 8.0), 150 mM NaCl, 0.5 mM EDTA, 5 mM MgCl_2_ and 1 mM ATP. For the auto-dephosphorylation process of KaiC, a solution containing KaiC alone was transferred from an ice bath to 30°C. The KaiC phosphorylation cycle was reconstructed *in vitro* by mixing KaiA (0.04 mg/ml), KaiB (0.04 mg/ml) and KaiC (0.2 mg/ml) as reported previously [11,18]. Relative abundance of the phosphorylation state was analyzed by SDS–PAGE and quantified using LOUPE software [37]. The period lengths were estimated by fitting the time-evolution of the fraction of the phosphorylated KaiC to a single cosine function. We further tested how the estimated period length was influenced by extending the number of harmonics as a Fourier series up to third and confirmed that the estimated period length was essentially the same irrespective of the number of harmonics. *Q*_10_ value, the factor by which the cycle frequency (a reciprocal of the period length) is accelerated by raising the temperature from 30°C to 40°C, was determined from the slope of the Arrhenius plot as described previously [9].

### Crystallization of KaiC^F419W^

The crystal of KaiC^F419W^ was obtained using the vapor diffusion method. The purified KaiC^F419W^ was concentrated up to 3.5 mg/ml in a solution of 20 mM Tris–HCl (pH 8.0), 150 mM NaCl, 5 mM MgCl_2_, 1 mM DTT and 1 mM ATP. The sample solution was mixed at a 1 : 1 ratio with a reservoir solution of 100 mM Tris–HCl (pH 7.0), 1 M KCl, 0.7 M sodium/potassium tartrate, 1.8 M sodium acetate, and 5 mM AMP-PNP. Because the reservoir solution itself exhibited the cryoprotectant effect, the crystal was picked up from the crystallization drop and directly soaked into the liquid nitrogen for the diffraction experiment conducted at the cryo-temperature.

### Data collection and structure determination

X-ray diffraction data were collected on beamline BL44XU at SPring-8 (Harima, Japan). The crystal was mounted under a cryostream at 100 K during the X-ray radiation. Diffraction images were recorded using EIGER X 16M (DECTRIS) and processed with XDSGUI [38]. Initial phase was obtained by molecular replacement using the crystal structure of KaiC-ST deposited as 7DYJ [29] and MOLREP [39]. Refinement was carried out using Refmac5 [40] with the free-R flags transferred from 7DYJ. The model building was conducted with COOT [41], and graphic representations of the model were generated using PyMOL (Schrödinger). The statistics for the diffraction experiment and the refinement are listed in Table 1.

**Table 1.** Data collection and refinement statistics.

Protein	KaiC^F419W^-ST
*Data collection*	
Space group	*P*6_3_
Unit cell parameters	
* a*, *b*, *c* (Å)	95.3, 95.3, 180.7
*α*, *β*, *γ* (°)	90, 90, 120
Wavelength (Å)	0.9
Resolution range (Å)^a^	47.6–2.84
	(3.01–2.84)
Total reflections	234 324
Unique reflections	43 729 (6952)
Redundancy	5.4 (5.5)
Completeness (%)	99.4 (98.2)
*R*_merge_ (%)^b^	8.5 (91.5)
(*I*)/sigma(*I*)	12.2 (2.0)
*Model building*	
Molecular replacement	KaiC-ST (7DYJ)
Total atoms	6682
Protein	6537
Ligands	128
Water	17
*R*_work_ (%)^c^	28.3
*R*_free_ (%)^c^	32.5
R.M.S.D. from ideality	
Bond length (Å)	0.002
Bond angles (°)	1.2
Average B factors (Å^2^)	80.7
Ramachandran plot	
Most favored (%)	85.2
Allowed (%)	14.8
Disallowed (%)	0.1
*PDB code*	7WDC

a Values in parentheses are for the highest-resolution shell;

b *R*_merge_ = Σ|*I −  *|/Σ*I*, where *I* corresponds to the observed intensity of reflections.

c *R*_work, free_ = Σ|F_obs_| − |F_calc_|/Σ|F_obs_|. *R*_free_ is the cross-validation of R-factor using the test reflections, 5% of the data, not included in the refinements.

### Fluorescence measurements

Fluorescence measurements were carried out at 30°C and the temperature was controlled with a precision of 0.1°C by using a LTB-125 water-bath (AS ONE Corporation). Fluorescence emission spectra from Trp residues were collected every 1 nm with a 0.5 s response time and a scan speed of 240 nm min^−1^ at an excitation wavelength of 295 nm (Hitachi, F-7000). The spectral bandwidth was set at 1.0 nm for excitation and 5.0 nm for emission. The observed spectra were normalized against both KaiC concentration and the fluorescence signal of an N-acetyl-l-tryptophan amide (NATA) standard solution with an absorbance of 0.05 at 280 nm. For static measurements, each sample was stored on ice, transferred to 30°C, and then incubated 10 min before measurements.

### Data analysis

The time-courses of fluorescence intensity during the auto-dephosphorylation process of KaiC alone were analyzed using the following equation [27,28]:

1 $$F_{\mathrm{app}}(t)=F_{\mathrm{SpT}}A_{\mathrm{SpT}}(t)+F_{\mathrm{pSpT}}A_{\mathrm{pSpT}}(t)+F_{\mathrm{pST}}A_{\mathrm{pST}}(t)+F_{\mathrm{ST}}A_{\mathrm{ST}}(t)$$

where *A_i_*(*t*) is the relative phosphorylation-state abundance (*i* = SpT, pSpT, pST, ST) pre-determined experimentally (blue circles, red triangles, green squares and orange diamonds in Figure 4A,B. The *F*_i_ values were estimated by least-square fitting of *F*_app_(*t*) to equation (1).

## Data Availability

All original data included in this paper are available from the authors upon reasonable request. Atomic co-ordinate and structure factor have been deposited in the Protein Data Bank with the accession codes: 7WDC (KaiC^F419W^) [42].

## Abbreviations

AMP-PNP: adenylyl-imidodiphosphate
ATP: adenosine triphosphate
DTT: dithiothreitol
EDTA: ethylenediaminetetraacetic acid
RMSD: root mean square deviation
SDS–PAGE: sodium dodecyl sulfate polyacrylamide gel electrophoresis
Tris: tris(hydroxymethyl)aminomethane
