# Supplementary material for: Highly sensitive tryptophan fluorescence probe for detecting rhythmic conformational changes of KaiC in the cyanobacterial circadian clock system
Source: Biochem J. 2022 Jul 19;479(14):1505–15. doi: 10.1042/BCJ20210544 (PMC9342895; doi:10.1042/BCJ20210544)
Supplement: Supplementary Material 1 [file BCJ-479-1505-s1.pdf]

Supplementary Material

```
CE         gagctcAGCTGCTCCGCAATGCCTATTCATGGGGAATGGACTTTGAGGAGATGGAGCGCC
EE         gagctcAGCTGCTCCGCAATGCCTATTCATGGGGAATGGACTTTGAGGAGATGGAGCGCC
EV         gagctcAGCTGCTCCGCAATGCCTATTCATGGGGAATGGACTTTGAGGAGATGGAGCGCC
CV         gagctcAGCTGCTCCGCAATGCCTATTCATGGGGAATGGACTTTGAGGAGATGGAGCGCC
F419W      gagctcAGCTGCTCCGCAATGCCTATTCATGGGGAATGGACTTTGAGGAGATGGAGCGCC
F419W-CE   gagctcAGCTGCTCCGCAATGCCTATTCATGGGGAATGGACTTTGAGGAGATGGAGCGCC
F419W-EE   gagctcAGCTGCTCCGCAATGCCTATTCATGGGGAATGGACTTTGAGGAGATGGAGCGCC
F419W-EV   gagctcAGCTGCTCCGCAATGCCTATTCATGGGGAATGGACTTTGAGGAGATGGAGCGCC
F419W-CV   gagctcAGCTGCTCCGCAATGCCTATTCATGGGGAATGGACTTTGAGGAGATGGAGCGCC

CE         AAAACCTCCTCAAAATTGTTTGCGCCTATCCTGAATCTGCAGGTCTTGAAGACCATTTGC
EE         AAAACCTCCTCAAAATTGTTTGCGCCTATCCTGAATCTGCAGGTCTTGAAGACCATTTGC
EV         AAAACCTCCTCAAAATTGTTTGCGCCTATCCTGAATCTGCAGGTCTTGAAGACCATTTGC
CV         AAAACCTCCTCAAAATTGTTTGCGCCTATCCTGAATCTGCAGGTCTTGAAGACCATTTGC
F419W      AAAACCTCCTCAAAATTGTTTGCGCCTATCCTGAATCTGCAGGTCTTGAAGACCATTTGC
F419W-CE   AAAACCTCCTCAAAATTGTTTGCGCCTATCCTGAATCTGCAGGTCTTGAAGACCATTTGC
F419W-EE   AAAACCTCCTCAAAATTGTTTGCGCCTATCCTGAATCTGCAGGTCTTGAAGACCATTTGC
F419W-EV   AAAACCTCCTCAAAATTGTTTGCGCCTATCCTGAATCTGCAGGTCTTGAAGACCATTTGC
F419W-CV   AAAACCTCCTCAAAATTGTTTGCGCCTATCCTGAATCTGCAGGTCTTGAAGACCATTTGC

CE         AGATTATTAAGTCGGAGATCAATGACTTTAAGCCAGCTCGTATTGCAATCGACTCCCTCT
EE         AGATTATTAAGTCGGAGATCAATGACTTTAAGCCAGCTCGTATTGCAATCGACTCCCTCT
EV         AGATTATTAAGTCGGAGATCAATGACTTTAAGCCAGCTCGTATTGCAATCGACTCCCTCT
CV         AGATTATTAAGTCGGAGATCAATGACTTTAAGCCAGCTCGTATTGCAATCGACTCCCTCT
F419W      AGATTATTAAGTCGGAGATCAATGACTTTAAGCCAGCTCGTATTGCAATCGACTCCCTCT
F419W-CE   AGATTATTAAGTCGGAGATCAATGACTTTAAGCCAGCTCGTATTGCAATCGACTCCCTCT
F419W-EE   AGATTATTAAGTCGGAGATCAATGACTTTAAGCCAGCTCGTATTGCAATCGACTCCCTCT
F419W-EV   AGATTATTAAGTCGGAGATCAATGACTTTAAGCCAGCTCGTATTGCAATCGACTCCCTCT
F419W-CV   AGATTATTAAGTCGGAGATCAATGACTTTAAGCCAGCTCGTATTGCAATCGACTCCCTCT

CE         CTGCTTTGGCGCGGGGCGTTAGCAACAATGCCTTCCGCCAATTTGTAATTGGTGTCACTG
EE         CTGCTTTGGCGCGGGGCGTTAGCAACAATGCCTTCCGCCAATTTGTAATTGGTGTCACTG
EV         CTGCTTTGGCGCGGGGCGTTAGCAACAATGCCTTCCGCCAATTTGTAATTGGTGTCACTG
CV         CTGCTTTGGCGCGGGGCGTTAGCAACAATGCCTTCCGCCAATTTGTAATTGGTGTCACTG
F419W      CTGCTTTGGCGCGGGGCGTTAGCAACAATGCCTTCCGCCAATTTGTAATTGGTGTCACTG
F419W-CE   CTGCTTTGGCGCGGGGCGTTAGCAACAATGCCTTCCGCCAATTTGTAATTGGTGTCACTG
F419W-EE   CTGCTTTGGCGCGGGGCGTTAGCAACAATGCCTTCCGCCAATTTGTAATTGGTGTCACTG
F419W-EV   CTGCTTTGGCGCGGGGCGTTAGCAACAATGCCTTCCGCCAATTTGTAATTGGTGTCACTG
F419W-CV   CTGCTTTGGCGCGGGGCGTTAGCAACAATGCCTTCCGCCAATTTGTAATTGGTGTCACTG

CE         GCTACGCGAAACAAGAAGAAATCACGGGACTATTCACAAATACCAGTGATCAATTTATGG
EE         GCTACGCGAAACAAGAAGAAATCACGGGACTATTCACAAATACCAGTGATCAATTTATGG
EV         GCTACGCGAAACAAGAAGAAATCACGGGACTATTCACAAATACCAGTGATCAATTTATGG
CV         GCTACGCGAAACAAGAAGAAATCACGGGACTATTCACAAATACCAGTGATCAATTTATGG
F419W      GCTACGCGAAACAAGAAGAAATCACGGGACTATTCACAAATACCAGTGATCAATGGATGG
F419W-CE   GCTACGCGAAACAAGAAGAAATCACGGGACTATTCACAAATACCAGTGATCAATGGATGG
F419W-EE   GCTACGCGAAACAAGAAGAAATCACGGGACTATTCACAAATACCAGTGATCAATGGATGG
F419W-EV   GCTACGCGAAACAAGAAGAAATCACGGGACTATTCACAAATACCAGTGATCAATGGATGG
F419W-CV   GCTACGCGAAACAAGAAGAAATCACGGGACTATTCACAAATACCAGTGATCAATGGATGG

CE         GAGCGCATTCGATTACTGACTCCCATATCTGTGAGATTACGGATACGATTATCTTGCTCC
EE         GAGCGCATTCGATTACTGACTCCCATATCGAAGAGATTACGGATACGATTATCTTGCTCC
EV         GAGCGCATTCGATTACTGACTCCCATATCGAGGTGATTACGGATACGATTATCTTGCTCC
CV         GAGCGCATTCGATTACTGACTCCCATATCTGTGTGATTACGGATACGATTATCTTGCTCC
F419W      GAGCGCATTCGATTACTGACTCCCATATCTCAACAATTACGGATACGATTATCTTGCTCC
F419W-CE   GAGCGCATTCGATTACTGACTCCCATATCTGTGAGATTACGGATACGATTATCTTGCTCC
F419W-EE   GAGCGCATTCGATTACTGACTCCCATATCGAAGAGATTACGGATACGATTATCTTGCTCC
F419W-EV   GAGCGCATTCGATTACTGACTCCCATATCGAGGTGATTACGGATACGATTATCTTGCTCC
F419W-CV   GAGCGCATTCGATTACTGACTCCCATATCTGTGTGATTACGGATACGATTATCTTGCTCC

CE         AATACGTCGAGATTCGTGGCGAAATGTCCCGCGCCATTAACGTCTTCAAGATGCGCGGAT
EE         AATACGTCGAGATTCGTGGCGAAATGTCCCGCGCCATTAACGTCTTCAAGATGCGCGGAT
EV         AATACGTCGAGATTCGTGGCGAAATGTCCCGCGCCATTAACGTCTTCAAGATGCGCGGAT
CV         AATACGTCGAGATTCGTGGCGAAATGTCCCGCGCCATTAACGTCTTCAAGATGCGCGGAT
F419W      AATACGTCGAGATTCGTGGCGAAATGTCCCGCGCCATTAACGTCTTCAAGATGCGCGGAT
F419W-CE   AATACGTCGAGATTCGTGGCGAAATGTCCCGCGCCATTAACGTCTTCAAGATGCGCGGAT
F419W-EE   AATACGTCGAGATTCGTGGCGAAATGTCCCGCGCCATTAACGTCTTCAAGATGCGCGGAT
F419W-EV   AATACGTCGAGATTCGTGGCGAAATGTCCCGCGCCATTAACGTCTTCAAGATGCGCGGAT
F419W-CV   AATACGTCGAGATTCGTGGCGAAATGTCCCGCGCCATTAACGTCTTCAAGATGCGCGGAT

CE         CTTGGCATGACAAAGCAATCCGCgaattc
EE         CTTGGCATGACAAAGCAATCCGCgaattc
EV         CTTGGCATGACAAAGCAATCCGCgaattc
CV         CTTGGCATGACAAAGCAATCCGCgaattc
F419W      CTTGGCATGACAAAGCAATCCGCgaattc
F419W-CE   CTTGGCATGACAAAGCAATCCGCgaattc
F419W-EE   CTTGGCATGACAAAGCAATCCGCgaattc
F419W-EV   CTTGGCATGACAAAGCAATCCGCgaattc
F419W-CV   CTTGGCATGACAAAGCAATCCGCgaattc
```

Supplementary Figure S1. **The synthesized gene sequences.** Codons that correspond to W419 substitution and phospho-mimicking mutations are highlighted in red and yellow, respectively. Bases marked with lower case letters represent recognition sites.
